# The workflow of single-cell expression profiling using quantitative real-time PCR

**DOI:** 10.1586/14737159.2014.901154

**Published:** 2014-03-21

**Authors:** Anders Ståhlberg, Mikael Kubista

**Affiliations:** ^a^ ^1^Department of Pathology, Sahlgrenska Cancer Center, University of Gothenburg, Box 425, 40530 Gothenburg, Sweden; ^b^ TATAA Biocenter, Odinsgatan 28, 41103 Gothenburg, Sweden; ^c^ Institute of Biotechnology, Academy of Sciences of the Czech Republic, Videnska 1083, Prague 4, 14221 Czech Republic

**Keywords:** gene expression profiling, preamplification, RT-qPCR, single-cell analysis, single-cell biology, single-cell workflow

## Abstract

Biological material is heterogeneous and when exposed to stimuli the various cells present respond differently. Much of the complexity can be eliminated by disintegrating the sample, studying the cells one by one. Single-cell profiling reveals responses that go unnoticed when classical samples are studied. New cell types and cell subtypes may be found and relevant pathways and expression networks can be identified. The most powerful technique for single-cell expression profiling is currently quantitative reverse transcription real-time PCR (RT-qPCR). A robust RT-qPCR workflow for highly sensitive and specific measurements in high-throughput and a reasonable degree of multiplexing has been developed for targeting mRNAs, but also microRNAs, non-coding RNAs and most recently also proteins. We review the current state of the art of single-cell expression profiling and present also the improvements and developments expected in the next 5 years.

## Why single-cell profiling?

**Figure 1.**
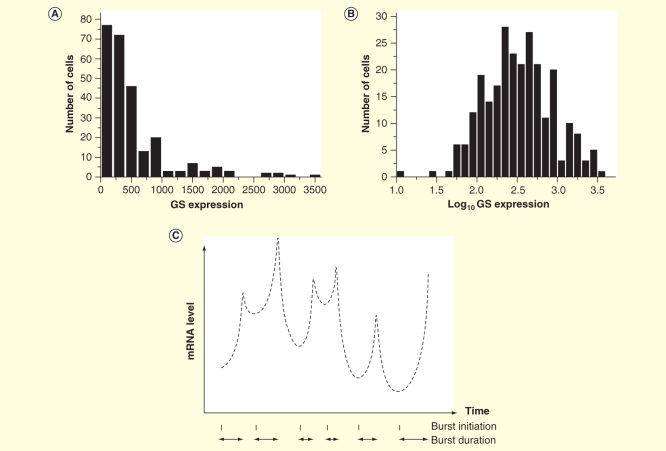
**Single-cell gene expression data. (A)** Distribution of transcripts among like cells is skewed **(B)** and can be modeled with avlognormal distribution [1], here, exemplified by the expression of GS in 258 primary astrocytes [51]. **(C)** Transcripts are produced in bursts, with variable frequency and amplitude [6]. The burst kinetic accounts for the lognormal distribution of transcripts among like cells.

Cytomics is the analysis of cell system (cytome) heterogeneity and the use of the measured data to determine the system’s molecular phenotype that results from its genotype and the exposure to environment [1]. Tissues comprises many cell types, often with specialized functions, which respond to different stimuli. If we are interested how an organ reacts to a change in environmental conditions, stimuli or a certain treatment, studying a traditional sample comprising hundreds of thousands of cells, then we measure the combined response of all the cells present. If only some cells, perhaps a minority cell type, are affected, then their response may go unnoticed against the background of all the nonresponsive cells. Disintegrating the tissue into individual cells that are sorted and then profiled one by one, we can much more sensitively detect and in much greater detail study the response. Also seemingly, homogeneous cells can show highly variable response to stimuli. This was demonstrated already in one of the first single-cell reverse transcription quantitative PCR (RT-qPCR) expression profiling papers in 2005, where highly skewed distribution of transcripts among seemingly homogeneous beta cells collected from a cell line was found [2]. The skewed distribution could be satisfactory modeled with a log normal distribution (Figure 1). Same kind of distribution was observed for all the transcripts studied and was also found in primary beta cells collected from the islets of Langerhan in mice. This skewed distribution has then been found for all transcripts in all kinds of cells that metabolize mRNA, suggesting that it reflects a fundamental behavior. Only known exception is the amphibian oocyte. They do not metabolize RNA and are very homogeneous as to the content of mRNAs. Studies of expression dynamics in individual cells using fluorescent probes have revealed a plausible mechanism. Expression takes place in bursts, with very rapid increase of the amount of a particular mRNA followed by a slow decay (Figure 1C) [3,4]. Currently, there are no mechanisms known that would synchronize bursts in individual cells. Integrating the burst kinetics over the cell population, a distribution of transcripts among cells that is consistent with the observed log normal distribution in single-cell profiling is obtained [4]. Recent theoretical studies suggest that more appropriate description might be the related gamma distribution, but with current measurement precision, the lognormal fitting commonly used is good enough [5]. The frequency of transcriptional bursts varies among genes and is typically in the order of minutes to hours [6]. Also proteins are produced in bursts, although the kinetics is slower, with a reported frequency of hours to days [7].

GS: Glutamine synthase.

When a traditional many-cell sample is studied, the total number of transcripts is measured. If we divide by the number of cells, then we obtain the normal, so called arithmetic average (e.g., the arithmetic average of 2 and 8 is: (2 + 8)/2 = 5). The arithmetic average, however, is not the expected count of transcripts in the typical cell of the sample. The statistical definition of the typical cell is the median cell, when the cells are sorted based on the number of the particular transcript they contain. Because of the underlying lognormal distribution, the number of transcripts in the typical cell will rather be the geometric average of the number of transcripts in each cell. The geometric average is obtained by multiplying the numbers of transcripts in each cell and then taking the nth root of the product (e.g., the geometric average of 2 and 8 is: √2 × 8 = 4). The geometric average is always lower than or equal to the arithmetic average; it can never be higher. Nor can the geometric average be determined from traditional studies of many-cell samples. It can only be calculated from single-cell measurements.

Most genes in a cell are expressed seemingly independently of each other, and the transcript levels measured across individual cells do not correlate. But there are exceptions. Genes involved in the same pathway or those that are part of a common network show correlated expression on the single cell level. Correlations of transcript levels are also seen on traditional many-cell samples. These correlations are exploited in diagnostics as expression signatures reflecting disease state, indicating response to treatment or predicting survival. Although these correlations are most powerful to predict clinical responses, they only reflect the genes that are affected by a certain environmental condition. The genes do not have to be, and usually are not, involved in the same biological process.

In this review, we discuss the experimental workflow of single-cell expression profiling. The rationale of each step is general for most single-cell methods, and we have chosen to exemplify them on the basis of qPCR methodology. For technology overviews, we refer to other reviews [8–13].

## Collecting single cells

Arguably, the most challenging step in single-cell profiling is to obtain representative individual cells with unperturbed expression profiles. Analysis of individual cells requires tissues to be dissociated. Cells are commonly separated from each other by mechanical forces, enzymatic digestion or a combination of both [14]. The generation of single-cell suspension is often accompanied by cell death and altered gene expression. Even established cell lines are affected by the enzymatic treatments [Ståhlberg A, Unpublis hed Data]. The bias induced by the cell dissociation depends on the protocol used, but it also affects the genes differently. So far, most single-cell studies ignore the bias introduced by the cell dissociation step. It is one of the most challenging steps to control, and more studies addressing cell dissociation are needed to elucidate its importance and effect on downstream applications [15]. Expression bias induced by sampling and preanalytical processing is a problem not exclusive to single-cell studies; it is a serious problem of all molecular diagnostics [16].

Methods such as FISH [17], *in situ* proximity ligation assays (PLAs) [18], spatial sequencing and microdissection [19] do not require cell dissociation. Information about the spatial position of each cell and its relation to different morphological parameters is often valuable information when interpreting the measured molecular signatures of individual cells. A drawback is that *in situ* analysis is hard to correlate to features of the individual cells, since cell borders are often difficult to identify and tissue preparations may cut through cells. Another limitation of *in situ* methods is that they require some cell fixation, which usually has negative impact on the nucleic acids’ integrity. Samples collected with microdissection for downstream analysis have similar limitations as the *in situ* methods.

A common way to collect cells today is by FACS. FACS has the advantage that cells can be selected for analysis based on light scattering and fluorescence, which reflect size, granulation, the presence of unspecific fluorescent markers and the specific binding of fluorescent labeled antibodies. These options to enrich for the cells of interest and the high-throughput capacity of FACS make it most useful for the screening of high cell numbers. The limitation is that the cells must be in suspension, which requires tissue to be dissociated and, consequently, the loss of the cells’ history. Another issue is cells are stressed, which may affect their expression profile. Also it is not possible to inspect the cells visually to decide which to collect. The development of QuantiGene FlowRNA and SmartFlare RNA detection probes is the two strategies that can detect and quantify specific RNAs using FACS, where the latter is applied on living cells [20,21]. DEPArray is a new technology that allows cells to be sorted in a similar way as by FACS, but allows for visual inspection and induces less stress [22].

A third strategy is to pick cells either manually or automatically using microaspiration technique [1,8]. Either the whole cell or the cytoplasm only is collected. The latter can be used when analyzing cells in tissue minimizing the perturbation caused by dissociation. However, when collecting cytoplasm, it is hard to control how much of the cytoplasm is extracted, which may introduce some variation. Advantage of microaspiration is that it is readily combined with visual inspection of the cytoplasm with essentially any microscopy setup.

The risk that tissue dissociation or general removal of the cells from their natural environment induces expression artifacts calls for proper controls. Generally, it is hard to prove that the collected cells represent the population of interest and that the measured profiles indeed reflect the *in vivo* expression. When all cells in a tissue are collected, one test is to sum the measured transcripts in all the cells and compare with the profile measured by traditional means of a corresponding many-cell sample. Agreement is expected to be high [23]. Disagreement would suggest that the particular protocol used for the collection of the individual cells introduces bias. This is most relevant control, but is only applicable when a dominant cell type is of interest. Any bias induced in a minority cell type will be masked by the expression of the dominant cells in the classical analysis. Another approach to validate the cell collection procedure is to apply two independent techniques and compare the outcome. New approaches to collect and/or enrich for specific cells are being developed [11,13] including label-free techniques such as acoustophoresis [24].

## Sampling ambiguity

**Figure 2.**
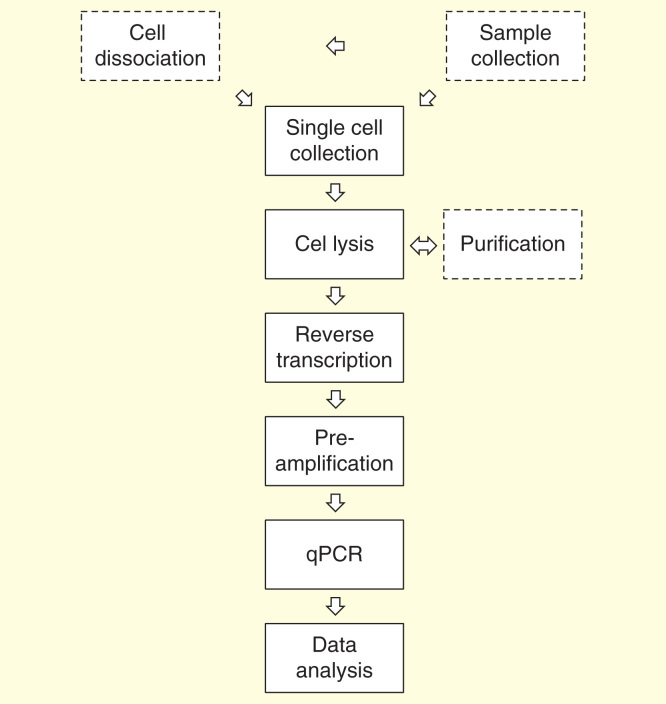
**Overview of single-cell gene expression profiling by real-time quantitative PCR.** Sample collection, cell dissociation and purification are study dependent steps.

**Figure 3.**
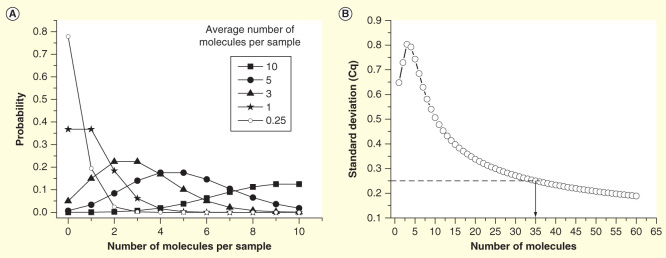
**Sampling ambiguity due to Poisson distribution. (A)** The probability to obtain a certain number of target molecules in an aliquoted volume, when the sample (average) concentration is 10, 5, 3, 1 and 0.25 target molecules per volume. **(B)** Expected standard deviation of Cq values of replicates as function of the average number of molecules per reaction volume. Off-scale data are ignored, which produces a maximum around three molecules. For comparison, SD of a typical qPCR within its dynamic range is indicated (SD = 0.25 cycles).

Because of the highly skewed (lognormal) distribution of transcripts among cells, even high expressed genes will have rather few transcripts in most cells. When analyzing single cells, it is important to use a workflow that minimizes losses (Figure 2). Optimally, this is a workflow without any washing steps, which inevitably lead to losses. These workflows are based on lysis reagents that keep the RNA intact and available and are compatible with downstream reverse transcription (RT) and subsequent PCR. After direct lysis, the RNA is reverse transcribed. RT yields vary; a range of 0.5–80% was measured for various target genes when the reverse transcriptase and the priming strategy were varied [25,26]. For single-cell work, it is critical to use a highly efficient reverse transcriptase that is not inhibited by the direct lysis reagent. The cDNA produced by the RT can be quantified directly by qPCR. However, if expression of multiple genes shall be measured, then preamplification should be considered, since it may improve precision. In profiling, qPCR is run in singleplex reactions: the sample is aliquoted, and one target is quantified per aliquot. Even if the qPCR assay is highly optimized and accurately measured, the number of target molecules in that particular aliquot, the approach may introduce very high confounding variation due to sampling ambiguity if the average number of targets per aliquot is low. Assume we are interested in analyzing 100 genes (in practice, it would probably be 96, but we assume 100 for simplicity). We also assume that the RT produces 100 cDNAs of a particular targeted transcript. If we divide the cDNA into 100 aliquots for singleplex qPCR, then we expect each aliquot to have in average one of the particular targeted cDNAs. In practice, each aliquot will not obtain exactly one target cDNA; rather there will be variation in the number of target cDNAs among the aliquots due to random effects of the sampling. Some aliquots will indeed contain a single target cDNA, but some will contain two, perhaps three or even more of the targeted cDNA, while some aliquots will have none. The probability an aliquot contains a particular number of target cDNA is given by the Poisson distribution, which for some selected cases are plotted in Figure 3A. For the case when the average concentration is one target cDNA per reaction volume, the probability to obtain exactly one target cDNA in an aliquot is 37%. It is 18% probability to obtain two, 7% to obtain three, but it is also 37% probability that an aliquot has none of the targeted cDNAs. From the latter, we calculate that the probability an aliquot taken from a sample containing on average one targeted cDNA per aliquoted volume is positive to: 100–37 = 63%. Corresponding calculation can be made for other average concentrations to produce a plot of the probability that an aliquot is positive versus the average concentration. From such plot, the theoretical limit of detection (LoD) of qPCR can be determined. If we analyze data and draw conclusions at 95% CI, then the LoD is the concentration at which 95% of the reads are positive. From the plot in Figure 3A follows, this is at an average concentration of three target molecules per reaction volume. In practice, because of limited RT efficiency, experimental impression and other confounding contributions, the LoD of an RT-qPCR analysis can be substantially higher.

qPCR: Quantitative PCR.

Sampling ambiguity also compromises the precision. The plot in Figure 3B shows the standard deviation (SD) of measured Cq values of replicates introduced by the sampling ambiguity [27]. It follows that the sample should have an average of some 35 target molecules per reaction volume to keep the contribution to SD from sampling ambiguity below 0.25 cycles, which in many studies would contribute significantly to the total confounding variance of the experiment [28]. If the single-cell content is divided into 100 aliquots, then the number of target mRNA molecules in the cell should have been 3500, assuming 100% RT efficiency, not to exceed this contribution. In reality, RT efficiency is limited [25,26], and a larger number is required. Because of the underlying lognormal distribution of transcripts among individual cells, only the most abundant transcripts will be present at 3500 or more copies in the majority of cells to be measured with reasonable accuracy based on a strategy that divides the original cell content into aliquots for singleplex qPCR, and even for those transcripts, many cells will have too small a number of mRNAs to be quantified with precision.

## Preamplification

Superior strategy to quantify many transcripts in a single cell is to perform RT on the total amount of extracted material and then preamplify the cDNA produced. Although several preamplification strategies have been described in literature, for single-cell profiling, the preferred method is target-specific multiplex PCR. The purpose of the preamplification (also known as PreAmp or target specific amplification) is to multiply the number of copies of the targeted transcripts such that the sample can be aliquoted for singleplex PCR without introducing serious sampling ambiguity. Critical, of course, is that the preamplification step itself does not introduce substantial variation or bias. It is well known that multiplex PCR is a highly complex reaction, where the simultaneous amplifications of the large number of targets may interfere. As amplicons from the most abundant target accumulate, their continued amplification consumes large amounts of reagents, which are depleted, compromising the PCR efficiencies and introducing bias. To avoid the depletion of reagents, preamplification should be run a limited number of cycles such that high level of any amplicon is avoided. Also, high-abundant targets, such as ribosomal RNAs, should not be included in the preamplification.

Most critical for successful preamplification is to use highly optimized qPCR assays. We typically aim to reach PCR efficiencies of ≥90% with high reproducibility (i.e., low random noise, also reflected by a narrow confidence interval of the estimated PCR efficiency). Many off-the-shelf assays offered commercially do not meet these criteria although they may perform satisfactory based on the criteria set up by the supplier. It is therefore advisable for designing our own assays, or order customized assays for high-performance qPCR by specialized providers. Using probe-based assays is an advantage since they usually perform better, and signals from aberrant products are suppressed. The probes are only used in the downstream qPCR; in the preamplification, they are left out or ignored. Nested designs can be used, which have the advantage that primer–dimer products formed during preamplification will not be amplified by the inner primers used in the downstream singleplex qPCRs. Another design strategy that may improve preamplification performance is to design all primers with 3′-termini that cannot interact in any combination; for example, all primers ending with either A-3′ or C-3′ [29]. It has also been suggested to treat the preamplified cDNA with Exonuclease I to remove unincorporated primers before proceeding with the singleplex qPCR [29].

The number of preamplification cycles needed depends on the downstream qPCR platform used and is mainly determined by its reaction volume. It also depends on the initial cDNA/DNA concentration, which may vary across cell types, but is primarily determined by the various dilution factors and volumes transferred in the workflow: the amount of mRNA transferred into RT; the fraction of the cDNA used for preamplification and the fraction of the preamplified cDNA transferred into each singleplex qPCR. If the preamplified material is divided into 96 singleplex qPCRs, then one more amplification cycle is needed than if it is divided into 48 aliquots only. Since the amount of preamplified material loaded onto the qPCR platform is small, then it is advantageous to keep reaction volumes down and concentrations high. This requires using reagents that are compatible. The direct lysis reagent may inhibit RT, and the cDNA reaction mix may inhibit PCR. Some suppliers have started to provide five-times reverse transcriptase reagents which have the advantage that a smaller volume is added compared with when using the traditional two-times mix.

Among the current high-throughput platforms, smallest reaction volume (6.75 nl) is used in the BioMark 96.96 dynamic arrays (for comparison of reaction volumes in high-throughput qPCR platforms, [30],). Out of this, about 40% originates from the preamplification mix; rest is added reagents, primers and reaction/loading buffers. Using 50 µl preamplification, Fluidigm recommends 18 cycles preamplification for single-cell profiling (and 14 cycles for conventional profiling). This is minimum and often suboptimum. If the cell contained a single mRNA molecule that indeed is reverse transcribed into a cDNA molecule, then 18 cycles of preamplification, assuming 100% PCR efficiency, produces 2^17^ = 131,000 copies (since cDNA is single stranded, the first PCR cycle does not amplify; it produces double-stranded cDNA, [25],). This gives an average of (0.4 × 0.00675/50) × 131,000 = 7 target amplicons per reaction chamber. An average of 7 is associated with substantial SD (Figure 3). In practice, it is worse because preamplification PCR efficiencies are not close to 100%. Rather, they are in the best case around 90% assuming that the assays are well designed for the purpose, and more often around 80% if less optimized assays are used. With 80% efficiency, there is an average of only (0.4 × 0.00675/50) × (1 + 0.8)^17^ ≈ 1 target amplicon per reaction chamber, which is even below the LoD at 95% CI. With assays having an efficiency of 90%, an average of three target amplicons per reaction chamber is obtained from a single template cDNA, which is just at the LoD. Hence, a single target molecule in the cell will generally be detected with 18 cycles preamplification using a highly optimized assay, but the precision in the quantification will be poor. Precision can be improved by reducing the preamplification volume and running few more preamplification cycles. Using 20 µl reaction volume and preamplifying for 20 cycles, a single target cDNA produces an average of (0.4 × 0.00675/20) × (1 + 0.9)^19^ ≈ 27 target amplicons per reaction chamber, which is reliably detected and readily quantified with high precision (Figure 3). The OpenArray from Life Technologies uses reaction volumes of 33 nl, and 18 cycles in 50 µl preamplification are sufficient to quantify down to a single copy with high precision. The WaferGen SmartChip uses 100 nl and the Roche LC1536 uses 500–2000 nl reaction volumes and require even less extensive preamplification.

The preamplification is a critical step in the single-cell profiling workflow and shall be thoroughly validated [31]. This is done using a validation sample that contains all the targets at reasonably high concentrations. It can be a field sample or a cDNA library, but often these will not contain all the targets at sufficient concentrations. Better is then to base the validation sample on purified PCR products or synthetic targets. The validation sample is split into halves. One half is analyzed by singleplex qPCRs for all the targets. The second half is divided into (at least) triplicates that each is preamplified and then analyzed in singleplex qPCRs for all the targets. In parallel, a nontemplate control is analyzed following the same scheme. The nontemplate controls are inspected to make sure none of the reactions produces primer–dimer products at levels that would interfere with quantification. The assay performance is assessed by comparing the measured Cq values with and without preamplification. For unbiased preamplification, the same difference between Cq values with and without preamplification is expected for all the assays. Small deviations are acceptable if they are reproducible, since they will cancel in any relative comparisons, which is standard procedure when analyzing expression profiling data [32]. Reproducibility is more critical. It is assessed for each assay separately by calculating the SD of the preamplification replicates. High SD limits the ability to measure biological differences [33], and assays that show poor reproducibility in preamplification should be redesigned or not trusted for small differences. If the validation sample was a cDNA library, then one shall verify that the target cDNA was present in sufficient concentration before disqualifying an assay, since a low starting concentration would also lead to high SD because of the sampling ambiguity (Figure 3B).

## qPCR

After preamplification, the sample is divided into aliquots, using some automatic or robotic loading system that usually is platform dependent, and is analyzed in singleplex qPCRs. qPCR replicates are generally not performed, since they add to the cost of the experiment and do not really improve precision, since the reproducibility of qPCR generally is very high. Rather, qPCR replicates may compromise precision if the preamplified cDNA has to be divided into a larger number of aliquots, since this will increase sampling ambiguity. If there is space on the qPCR platform, then it is better to analyze more cells than running technical replicates [33]. The qPCR assays can be either dye or probe based. Probes have the advantage that the interference from primer–dimer signals is suppressed. However, Cq values of probe-based assays can be compromised by the presence of primer–dimer products even when these are not seen due to competition for reagents [34].

## Normalization & data analysis

Data should not be normalized. Rather, cells shall be compared based on the data as measured, which is equivalent to normalization per cell. This is the far most intuitive way to compare expression data for single cells. One should absolutely not normalize to any kind of house-keeping genes or presumed reference genes, since the burst kinetic described above gives rise to seemingly uncorrelated variations between randomly selected genes and any such normalization would mess up the data, resulting in gibberish [23].

For most cell types, the experimental protocol is highly reproducible, and corrections for yield variations are not needed [2,29,31]. Most challenging are cells with high lipid content, such as adipocytes and oocytes that typically require an extraction protocol based on washing, which may lead to losses. For those cells, the protocol should be validated using a spike, preferably an artificial RNA with A-tail and 5′-cap to mimic the behavior of native mRNA [35]. Optimally, the spike is microinjected into the cell, in which case, it reflects also the extraction yield.

Single-cell expression data are analyzed following essentially the same steps as for traditional data. Detailed step-by-step guide was recently published [27], and only the main aspects are discussed here. Single-cell expression data typically suffer from high level of missing or off-scale data, where off-scale data refer to Cq values too high to be trusted. When using dye reporter, off-scale data are usually due to the formation of aberrant PCR products known as primer–dimers and can be recognized by performing melt curve analysis. Those Cq values cannot be trusted and should be deleted. Missing data are then replaced for each gene separately for the highest trusted Cq value measured plus an offset. If the cells studied are of the same type and expected to express common markers, then a small offset such as +1 should be used, since the failure to record a Cq value in those cases most likely is due to that particular reaction chamber did not receive a target molecule. An alternative is to impute the missing data taking into account the expression level of the other genes [27]. If the sample is heterogeneous with respect to cell types or cell states characterized by the expression of specific markers, then a larger offset, such as 4–6, shall be used to give the missing marker a high significance. Data are typically autoscaled to give all the markers equal weight and analyzed with multivariate methods such as principal component analysis, Hierarchical Clustering and the Self-Organized Map [36]. Usually, the profiling, at least initially, is performed for a large number of markers many of which will not be responsive to the particular treatment or environmental conditions studied. Removing the nonresponsive markers will improve separation in the multivariate analyses. Interactive tools, such as dynamic principal component analysis, allows variable selection to be performed based on either the p-value or differential expression between two groups or based on the variance for any number of groups.

## Other biomolecule targets

Although mRNA is the common target in single-cell expression profiling, also miRNA can be profiled using the same workflow [37]. The reaction is not as specific as for mRNA and the sensitivity is lower, but this is due to general limitations when assaying a short template molecule and not particular to single-cell work. Proteins can also be profiled using PCR-based methods. Two related techniques, PLA and proximity extension assays, bind two oligo-tagged antibodies to the same protein [38,39]. The simultaneous binding brings the oligonucleotides into proximity, which makes template for PCR. Preamplification can be introduced into the workflow for the simultaneous analysis of large number of target proteins. Recently, qPCR, RT-qPCR and PLA were used to measure the amount of transfected DNA, mRNA, miRNA, long noncoding RNA and protein in the same single cell [40]. DNA modifications such as methylation are also possible to monitor with single-cell resolution [41]. Results were very encouraging, showing significant correlation between the cellular levels of related biomolecules, implicating that it shall be possible to map interactions and networks involving different biomolecules on the single cell level.

## Expert commentary

Today, several methods and workflows have been developed and applied to analyze individual cells. New techniques are continuously reported, all with their advantages and limitations. However, the number of comparative studies of different methods is small, and efforts to reproduce reported data hardly exist. Many single-cell profiling studies have been performed, several based on large numbers of cells, but usually only from a small number of biological samples. This precludes an evaluating of the biological significance of the reported findings. Handling and analyzing individual cells containing very few target molecules call for highly optimized and carefully validated experimental workflow that are reported in detail, including early steps such as sample selection and cell collection procedure, as well as data preprocessing and analysis. The minimum information for publication of quantitative real-time PCR experiments is one effort that has significantly improved the way qPCR experiments and data are reported, allowing for reliable conclusions to be drawn [42]. Open access to reported single-cell data will also help the single-cell profiling field to develop from being a tool for highly specialized laboratories into a standardized and robust platform.

## Five-year view

Single-cell expression profiling is truly enabling. We learn things about cells that cannot be deduced or calculated from bulk measurements, but can only be extracted from measurements on the individual cells. This will lead to new insights into biology, novel discoveries and possibly even challenge some dogmas. Particularly exciting will be the new possibilities to characterize cell types and study their differentiation and proliferation. The tens of trillions (10^13^) of cells in a human body are often said to be made up of 210 cell types subdivided into 20 categories assembled in 1989 based primarily on function [43]. A more recent classification suggests that there are 411 cell types [44]. However, a precise and unambiguous definition of cell type are notoriously difficult. Environmental conditions, external stimuli, number and nature of neighboring cells, signals from remote cells through hormones, exosomes and other signaling substances, access to nutrients, oxygen and other vital substances, removal of waste products, phase of cell cycle, accumulated somatic mutations, integrated viruses, transposons, epigenetic alterations, chromosomal rearrangements and perhaps even age and generation will affect a cell’s molecular activities. Some may lead to virtually irreversible differentiation, while other may lead to reversible or even temporal changes only. Single-cell profiling is expected to shed light on these processes, perhaps by identifying cell type-specific expression networks that will contribute to establishing a definition of cell type and defining the molecular events that make a change virtually irreversible.

Single-cell profiling will revolutionize the exploration of expression pathways, networks and biomolecular interactions. These are fields currently in rapid development, theoretically as well as experimentally. Today, this work is based on the profiling of traditional many-cell samples. A stimuli usually affects many pathways and a challenge in analysis is to separate all the affected biomolecules into distinct pathways and networks. Analyzing single cells is possible, indeed likely, that independent pathways will be affected in different cells, which makes deconvolution much easier, even trivial in some cases.

Imprinting, allelic discrimination and selective allele inactivation are biological phenomena that seem critical for normal development, and errors in allelic expression may cause disease, even cancer in some cases [45,46]. These phenomena are studied on traditional many-cell samples today, making it difficult to detect rare effects, such as the illegitimate activation of an allele in a minority of the cells. With single-cell profiling, using assays with single base discrimination, the differential activity of paternal and maternal alleles can be measured by taking advantage of single-nucleotide polymorphism. With next-generation sequencing (NGS) suitable single-nucleotide polymorphisms are readily identified by the sequencing of parental DNA. In fact, NGS is emerging as most valuable complement to qPCR for single-cell profiling. New methods for library preparation are being developed to preamplify the whole transcriptome [47]. The NGS workflow is less robust than RT-qPCR, suffering from greater bias and larger variation. There may even be some drop outs. But the whole transcriptome is analyzed, which is most valuable as an initial screen to identify the most relevant genes to be studied in greater detail, higher throughput and better precision by RT-qPCR. An exciting emerging platform for single-cell profiling is the nCounter Analysis System from Nanostring [48]. Barcoded probes are hybridized to targets and counted using single molecule imaging. Several hundreds of targets can be detected in a single reaction, which positions the nCounter in between RT-qPCR and NGS in multiplex capability. However, sensitivity is not sufficient for direct analysis of the transcripts in the single cell. Preamplification is needed, which, as in the cases of RT-qPCR and NGS, introduces bias and variation. All three methods include a RT step, which is known to be highly reproducible, but introduces gene-specific bias [25,26]. Since the bias is rarely (never) determined for all the transcripts studied; only relative comparisons are possible with these techniques. A most exciting new technology for single-cell profiling is being developed by Cellular Research [49]. It is based on the tagging of the transcripts with molecular labels of various sequences with a generic tag from a large pool. This makes most transcripts different, with a low probability that the two transcripts obtain the same label. After RT and PCR amplification using a gene specific and a generic primer, the number of molecular labels represented on each particular transcript is interrogated by hybridization. Correcting for Poisson distribution, like in digital PCR [50], this provides the absolute count of the number of transcripts that were present initially. Currently, this is the only technology that approaches the determination of the absolute count of the different transcripts in a cell.

## Notes

Key issues

- Tissues are heterogeneous, and even cells of the same type respond differently to stimuli. This is resolved with single-cell profiling.
- Single-cell collection often requires advanced sample preprocessing that may affect the measured expression profile, highlighting the need for controls.
- Sampling ambiguity introduced by the handling of few molecules (<25) is given by the Poisson distribution and is an issue in single-cell analysis.
- To minimize sampling ambiguity, the number of molecules processed should be maximized in all steps of the protocol including cell lysis, reverse transcription, preamplification and quantitative PCR.
- Profiles should be compared as measured per cell; normalization to house-keeping genes or other tentative reference genes introduces uncontrolled errors.
- Emerging technologies allow multianalyte (DNA, RNA and protein) analysis in the same cell.
- Single-cell expression profiling opens up new avenues in molecular biology and diagnostics including improved tools to define cell types, explore expression pathways and characterize expression networks
- Single-cell profiling makes it possible to characterize rare cells.
